# State and Local Health Departments: Research, Surveillance, and Evidence-Based Public Health Practices

**DOI:** 10.5888/pcd20.230142

**Published:** 2023-09-28

**Authors:** Sara L. Huston, Austin Porter

**Affiliations:** 1 Maine Center for Disease Control and Prevention, Augusta, Maine; 2 Muskie School of Public Service, University of Southern Maine, Portland, Maine; 3 Fay W. Boozman College of Public Health, University of Arkansas for Medical Sciences, Little Rock, Arkansas; 4 Arkansas Department of Health, Little Rock, Arkansas

## Introduction

Health departments from the 50 states and thousands of local health departments are critical components of the US public health infrastructure, serving as the “backbone” of the public health system (1). These agencies provide a wide range of much-needed services to the community, including screening for chronic health conditions, vaccine administration, and tobacco use cessation as well as working to address health inequities and social determinants of health. Health departments create systems change and policy change to support health and prevent and control chronic conditions, work to ensure health-promoting environments, and monitor diseases and risk factors. This special collection highlights the breadth of work taking place in state and local health departments to improve the health and well-being of their communities, with topics such as cardiovascular disease (CVD), cancer screening, infant and maternal health outcomes, human papillomavirus (HPV) vaccination, community–clinical linkages, and food insecurity. The manuscripts in this collection further our knowledge of public health practice implementation by state and local health departments, covering a diverse array of chronic disease outcomes within 4 overarching themes:

1. Public health surveillance, epidemiology, and geographic information science and technology (GIS&T)
2. Intervention programs and tools
3. Public health practice across health agencies
4. Collaborations and partnerships

## Public Health Surveillance, Epidemiology, and Geographic Information Science and Technology

Many of the articles in this collection highlight the wide range of robust public health surveillance and epidemiology work happening at state and local health departments, and several incorporate GIS&T methods. State health department staff in Mississippi present their Joinpoint regression analysis of trends in CVD mortality in their state, finding increases in the death rate for those aged 55 to 64 years (2). Increases or plateauing of CVD mortality trends are an important public health topic and have been noted elsewhere after decades of decline (3,4). Salahuddin et al used multivariate regression and GIS&T methods to examine maternal sociodemographic and pregnancy-related risk factors and infant mortality in 2 Texas counties, mapping these at the zip-code level and using findings to develop strategies and programs in these communities to address infant mortality (5).

Two articles focus on the development of epidemiologic tools and techniques that may be useful for others. Whitley et al (6) describe in detail their approach to creating health rankings for Philadelphia neighborhoods by using data from the 500 Cities Project (7) as well as locally accessible data; and Foraker et al describe their open-source tool that can allow health agencies to map COVID-19 (or other) cases to the street level and show geographic changes over time to better plan public health response (8).

DuClos et al describe Florida’s use of GIS&T methods to create an interactive web map identifying areas at high risk for serious COVID-19 illness (9), and the article by Askelson et al reports on Iowa’s effort to use GIS&T tools to map HPV vaccine initiation rates by zip code tabulation areas and identify demographic and socioeconomic factors associated with HPV vaccine initiation to better tailor vaccination efforts (10). These 2 articles also highlight the interconnected nature of chronic and infectious disease: HPV vaccination can prevent certain types of cancers, while chronic conditions can increase the risk of serious COVID-19 illness and vice versa. Finally, Brissette et al describe a unique collaboration between the US Centers for Disease Control and Prevention (CDC) and several state and local health departments to highlight how these health agencies are incorporating GIS&T into their chronic disease work and the benefits and challenges of this work (11).

## Intervention Programs and Tools

Intervention programs or tools developed by and for local and state health agencies is another major theme of this collection. These programs and tools can serve as models for other health agencies. Authors from the Washington State Department of Health report on their evaluation of a program providing “prescriptions” in the form of $10 vouchers for eligible low-income patients to purchase fresh fruits and vegetables at participating grocery stores (12). This program resulted in more than $150,000 worth of vouchers being redeemed and nearly 90% of survey respondents eating more fruits and vegetables because of the vouchers (12). Smith et al report on their work with Arkansas barber and beauty shops to provide chronic disease education and screening, resulting in more than 500 people referred for treatment; referred patients indicated overall improvements in chronic disease symptoms, showing the effectiveness of tailoring interventions for populations to connect with them where they are (13).

Two articles describe work with pharmacists to improve chronic disease outcomes. Ross et al report on their chronic disease management program in Mississippi where pharmacists met with underserved patients newly diagnosed with diabetes, hypertension, or high cholesterol to review medications, create medication action plans, and refer patients for follow-up care when necessary (14). This intervention resulted in significant improvements in blood pressure, cholesterol levels, glycated hemoglobin A_1c_ (HbA_1c_), and triglycerides. The article by Jakeman et al reports on their effort to build on existing partnerships with pharmacies around tuberculosis testing to also provide latent tuberculosis infection (LTBI) treatment, to improve the accessibility of that treatment (15). Three-quarters of LTBI patients chose to use a community pharmacy once that option was provided, and they achieved high treatment completion rates.

The Program Evaluation Brief from Juster et al describes the development, implementation, and evaluation of their media campaign designed to reach primary care providers (PCPs) and increase provider-assisted smoking cessation. They found that those providers who had seen a campaign advertisement were more likely to provide their patients with evidence-based cessation treatment (16). In their Tools for Public Health Practice article, Phillips et al present their work to increase cancer screening among state employees, which is a large population in many states, providing tools and strategies that can be used by other health agencies to conduct similar interventions (17).

Finally, 2 articles describe interventions from multiple health agencies. Linabarger et al report on the design and evaluation of projects in 6 state health departments to strengthen collaboration between state oral health and chronic disease programs, focusing on common risk factors. The article highlights barriers to and facilitators of implementation and provides a framework and ideas for collaboration topics that can be used by other health agencies (18). In their Program Evaluation Brief, Felipe et al describe and provide lessons learned from a learning collaborative that engaged 31 state and territorial health agencies to improve hypertension diagnosis and management, incorporating a systems-change approach and rapid quality improvement processes (19).

## Public Health Practice Across Health Agencies

Several of the articles in this collection examine practices across multiple health agencies, either nationwide or statewide, providing a snapshot of current practices and opportunities for improvement. Rodriguez Weno et al conducted a survey of local and state health departments representing all 50 states to assess their knowledge and use of The Community Guide, a free online resource highlighting evidence-based practices (20). The results of the survey indicate that 80.9% of state and 53.3% of local health departments use The Community Guide to inform their practices (20). Use of evidence-based practices not only helps increase effectiveness and improve health outcomes but also allows for the efficient use of resources.

Researchers from the University of Nebraska Medical Center conducted surveys and interviews to assess collaboration efforts between local health departments and primary care clinics in Nebraska (21). There were strong collaborative efforts with local health departments and primary care providers in several activities, including the National Diabetes Prevention Program, screening services, worksite wellness programs, and others (21). Through these partnerships, local health departments were able to optimize services to the community and establish reliable referral routes for those in need. Tool kits, publications, and other online resources can provide the latest and greatest evidence-based practices and competencies that can be used by state and local health departments to deliver effective and efficient services to the community, and the article by Kane et al describes the process of revising the Core Chronic Disease Prevention Competencies (22).

## Collaborations and Partnerships

Collaborations and partnerships are often needed to advance the field of public health practice. Nearly all of the articles featured in this collection have a common theme of collaborating with academic institutions, federal agencies such as CDC, community-based organizations, and private industry, and even collaboration between programs within health agencies. Local and state health departments have been able to leverage these partnerships to increase the reach and effectiveness of many public health programs and interventions (13,14,21,23). Minwoong Chung, a researcher at the University of Hawaii, partnered with the National Association of County and City Health Officials (NACCHO) and Michigan State University to determine the extent to which local health departments used an informal network to share information regarding public health practices (23). In efforts to improve the outcomes of patients who were recently diagnosed with a chronic health condition, the Mississippi Department of Health partnered with the University of Mississippi School of Pharmacy, CDC, and the Aaron E. Henry Community Health Services (14). The Arkansas Department of Health partnered with local barber and beauty shops to conduct screening programs for chronic conditions (13). Practitioners from the Florida Department of Health collaborated with CDC and the Council of State and Territorial Epidemiologists (CSTE) to examine severe maternal outcomes related to hypertension (24). Partnerships with organizations help strengthen the public health infrastructure that will ultimately improve the health and well-being of our communities.

## Gaps and Barriers

The literature is rich in providing evidence-based practices and interventions for health practitioners; however, journals capture only a small fraction of the evidence that is produced by programs and activities implemented by local and state health departments. State and local health departments are engaging in key efforts in areas such as understanding the impact of social determinants of health, mitigating health disparities, studying the long-term effects of COVID-19, and improving mental and maternal health. However, because of barriers and other restraints, much of this work is not published in peer-reviewed journals. There is a need to fill the gaps in the literature by highlighting the work that is conducted by local and state health departments. This will allow health practitioners and researchers to have access to a broader and deeper selection of evidence-based practices.

Oftentimes, health departments face certain barriers when trying to publish their work, including lack of time and resources. State and local health departments are at the forefront of many public health emergencies, including the COVID-19 pandemic and mpox outbreak. Because of this, these agencies may not have the necessary time to draft and submit peer-reviewed manuscripts for consideration. Pittman et al found that time was the greatest barrier to publication among applied epidemiologists (25). The process may take several months, and department leadership may not support allocating resources to this endeavor. Another potential barrier is not having a relationship with academic institutions. A survey of health departments conducted by Erwin et al found that only 55% of health departments identified as an “academic health department,” where health departments have established a formal and collaborative relationship with an academic institution (26). Being unfamiliar with the process of submitting to a peer-reviewed publication is another barrier that practitioners often face. In the same survey conducted by Pittman et al, not knowing how to submit papers was another barrier indicated by respondents (25). The process of preparing and submitting a manuscript can be challenging and even intimidating (27). Lastly, practitioners may not know which journal is appropriate for their work or may feel as though their manuscript is not “good enough” for publication. They may perceive that their work or intervention is of interest only to their local community (28). Peer-reviewed publications are effective means of highlighting evidence-based, promising, or emerging practices with wide audience reach that can inform state and local health departments.

## Recommendations

Our recommendations to improve the dissemination of state and local health department work through peer-reviewed publication fall into 4 interconnected areas: leadership and vision, collaboration, training and technical assistance, and visibility and incentives. Five key sectors all have influence and potential contributions in each of these categories. These sectors are public health journals, national organizations representing state and local health departments (such as the Association of State and Territorial Health Officials, NACCHO, National Association of Chronic Disease Directors, CSTE, and others), funding agencies (such as CDC, Health Resources and Services Administration, Substance Abuse and Mental Health Services Administration, and others), academic centers, and the state and local health departments themselves. We recognize that some of the work we are about to suggest is already ongoing; we are encouraging these efforts to be expanded, sustained over time, and institutionalized.

First, under leadership and vision, we call on leaders in these 5 sectors to promote peer-reviewed publications as an achievable dissemination method for state and local health departments, and to provide leadership, resources, and tools to make that vision a reality. Second, as the articles in this collection demonstrate, collaboration of state and local health department authors with authors from funding agencies, national organizations, and academic centers appears to accelerate dissemination of health department work through peer-reviewed publications. Public health faculty in academic centers may be especially important collaborators for state and local health departments given their expertise in manuscript publication and the publication expectations of their positions. We invite public health professionals in all sectors to be the first to reach out to others to collaborate on publications, and we encourage leaders in these sectors to build sustainable environments that facilitate collaboration over time. Potential efforts to bring writing collaborators together could include special events, dedicated time at standing meetings or conferences, and the formation of collaborative writing groups. Ideally, these efforts would have a longer-term vision and commitment such that they become institutionalized and viewed as business as usual.

Third, training and technical assistance are vitally important to ensuring that important work from state and local health departments is disseminated in the peer-reviewed literature. We suggest that journals interested in publishing more work from state and local health departments collaborate with funding agencies, academic centers, and the national organizations that represent health departments to provide periodic webinars or workshops to state and local health department staff on topics such as choosing the right journal and manuscript type, determining authorship and co-authorship, and navigating the peer-review process. Academic centers can also play a key role in ensuring training of the future public health workforce by increasing training and mentoring of bachelor’s-level and master’s-level public health students in peer-reviewed publication, as is the standard for doctoral students.

Fourth, we believe that increasing the visibility of and incentives for peer-reviewed publications by state and local health departments will normalize their publication, such that health department staff perceive this as a common and achievable method of disseminating health department work. Efforts here might include journals issuing a Call for Papers written by state and local health department first authors or in collaboration with health department staff, or special annual awards for the best articles authored by state and local health departments.

## Conclusion

State and local health departments are implementing important and effective public health programs that are worthy of dissemination in the peer-reviewed literature, where they may provide models for other health departments. The manuscripts featured in this collection and those published elsewhere are just a glimpse of the magnitude of efforts taking place within these agencies. In the wake of the COVID-19 pandemic and other public health events with national and global health implications, the need to highlight the work done by health departments is ever more urgent. While we acknowledge the barriers that exist in preparing manuscripts for submission, we hope that we have provided some actionable ideas to overcome those barriers and ensure more work from state and local health departments is disseminated in the peer-reviewed literature.
